# Supplementary material for: Population-Level Risk Factors Related to Measles Case Fatality: A Conceptual Framework Based on Expert Consultation and Literature Review
Source: Vaccines (Basel). 2023 Aug 21;11(8):1389. doi: 10.3390/vaccines11081389 (PMC10458804; doi:10.3390/vaccines11081389)
Supplement: Supplementary file 1 [file vaccines-11-01389-s001.zip › vaccines-2503564-supplementary.pdf]

Supplementary Information for Population-level risk factors related to measles case fatality: a conceptual framework based on expert consultation and literature review

Table of Contents

Section S1. Activities of the Expert Working Group **2**

Section S2. Full list of identified indicators potentially related to measles case fatality identified by Expert Working Group **4**

Section S3. Post-discussion list of identified indicators related to measles case fatality **5**

Section S4. Ranked list of indicators related to measles case fatality, with average rank. **6**

Section S5. PRISMA checklists **7**

Section S5a. PRISMA compliance checklist **7**

Section S5b. PRISMA compliance abstract checklist **11**

Section S6. Full list of indicator-specific search terms for systematic review **12**

Section S7. Studies containing evidence of an association between measles CFR and specified indicator **13**

Section S8. Studies containing non-significance evidence of an association between measles CFR and specified indicator **15**

Section S9. Indicators with significant evidence of an association with highest quality score from contributing studies **18**

Section S1. Activities of the Expert Working Group

The Working Group was established with the intent to gain feedback throughout the development of a conceptual framework as related to measles CFR. Members of the Working Group included:

- Natasha Crowcroft (WHO)
- Felicity Cutts (LSHTM)
- Emily Dansereau (BMGF)
- Matthew Ferrari (Penn State)
- Deepa Gamage (WHO)
- Katy Gaythorpe (VIMC)
- Kendall Krause (BMGF)
- Katrina Kretsinger (WHO / CDC)
- Kevin McCarthy (IDM / BMGF)
- Mark Papania (CDC)
- Niket Thakkar (IDM / BMGF)

The overall objectives for convening this Working Group, related to indicator investigation, were as follows:

1. Determine all possible indicators (and proxy metrics, as needed) as related to measles CFR
2. Determine relative order and group of available indicator importance
3. Provide guidance and recommendation on targeted literature reviews
4. Approve final indicator list and conceptual framework

Each session of the Working Group was conducted online, via Zoom. Activities for each Session are outlined below.

Session 1

Objectives

1. *Discuss a full list of possible covariates to explore, including proxies*
2. *Determine overall importance of each covariate candidate*

Working Group members brainstormed a comprehensive, full list of possible population-level indicators related to measles CFR. These were not to include those describing special populations, such as internally displaced persons and refugees, as the underlying available CFR data does not include adequate information on these groups. The full list of indicators generated by the Working Group are defined in Supplementary Information Section 2. Working Group members voted for each indicator that they thought had an important relationship with measles CFR; members could “up-vote” or “down-vote” for each. Indicators with no more than 2 down-votes were considered for further inclusion. This list can be found in Supplementary Information Section 3.

Session 2

Objectives:

1. *Anonymously rank indicators to determine relative importance*
2. *Determine indicator candidates further worth investigation*

Working Group members ranked indicators in order of importance to consider relative to one another. Each ranked position from 1 to 42 was assigned each corresponding weight. Age was removed from this process. Overall indicator rank was determined by average weight across responses, shown in Supplementary Section 4.

Members asserted, with at least one verbal yes, their desire for the inclusion of all indicators for further analysis. Members suggested considering mechanisms that might impact measles mortality or case fatality so it could be ensured that remaining indicators adequately captured the underlying components of these possible mechanisms.

Session 3

Objectives

1. *Review mechanistic groups and indicators per group*
2. *Review protocol for literature review and dataset investigation*

Members reviewed the following mechanistic groups and indicators corresponding with each group. The groups and related covariates are described in Table 1 (main text).

Members confirmed the inclusion of all indicators other than sanitation quality and the following protocol for literature review and data analysis:

1. Search for and review any available literature (systematic literature review)
2. Search for and review any available population level data (database search)
3. Categorize into following groups:
    a. Published literature supporting causal relationship and population-level data
    b. Published literature supporting observational relationship and population-level data
    c. Published literature with supporting qualitative evidence and population-level data
    d. No literature published, but population-level data available
    e. No literature published and population-level data is untrustworthy, contains missingness, or is otherwise unsuitable
4. Follow-up with Working Group to share indicator categories
5. Framework development

Session 4

Objectives

1. *Provide feedback on proposed conceptual framework of mechanistic groups*
2. *Review results from literature review and dataset investigation*
3. *Provide specific recommendation for areas in literature with ambiguous results*

From a systematic review of the literature, each covariate was classified in Table 2 (main text).

Section S2. Full list of identified indicators potentially related to measles case fatality identified by Expert Working Group

- Access to intensive care unit (ICU)
- Age
- Ambient air pollution
- Antibiotic use for measles-related pneumonia
- Asthma prevalence
- Autoimmune condition prevalence
- Average household size
- Bacille Calmette-Guérin vaccination coverage
- Breastfeeding prevalence
- Cancer prevalence
- De-worming frequency
- Diarrheal disease prevalence
- Diphtheria- tetanus- pertussis (DTP) vaccination coverage
- Educational attainment
- First-dose coverage of measles-containing vaccine (MCV1)
- Health expenditure per capita
- *Haemophilus influenzae* type B (Hib) vaccination coverage
- Human immunodeficiency virus (HIV) prevalence
- Human immunodeficiency virus (HIV) treatment / antiretroviral therapy (ART) prevalence
- Household air pollution
- Level of health care available
- Lower respiratory infection (LRI) prevalence
- Malaria prevalence
- Maternal antibody dynamics
- Maternal measles vaccination coverage
- Maternal smoking prevalence
- Measles attack rate / incidence
- Meningococcal serogroup A vaccination coverage
- Oral rehydration treatment or solution (ORT/S) for measles-related diarrhea
- Outbreak susceptibility
- Overweight prevalence
- Pneumococcal conjugate vaccination (PCV) coverage
- Polio vaccination coverage
- Pre-term birth prevalence
- Rotavirus vaccine coverage
- Rubella vaccine coverage
- Sanitation quality
- Second-dose coverage of measles-containing vaccine (MCV2)
- Sex
- Stunting prevalence
- Surrounding conflict
- Time
- Time to care seeking
- Total fertility rate
- Travel time to major city or settlement
- Travel time to nearest health care facility
- Tuberculosis prevalence
- Under-five mortality rate
- Underweight prevalence
- Vaccination efficacy
- Vaccination schedule
- Vaccine coverage equity
- Vitamin A deficiency prevalence
- Vitamin A supplementation prevalence
- Vitamin A treatment prevalence
- Wasting prevalence
- Water quality
- Yellow fever vaccination coverage

Section S3. Post-discussion list of identified indicators related to measles case fatality

- Access to ICU
- Age
- Ambient air pollution
- Antibiotic use for measles-related pneumonia
- Average household size
- Breastfeeding prevalence
- De-worming frequency
- Diarrheal disease prevalence
- Educational attainment
- Health expenditure per capita
- HIV prevalence
- HIV treatment prevalence / ART prevalence
- Level of health care available
- LRI prevalence
- Malaria prevalence
- Maternal antibody dynamics
- Maternal measles vaccination coverage
- Measles attack rate / incidence
- MCV1 coverage
- MCV2 coverage
- ORT/S for measles-related diarrhea
- Outbreak setting indicator
- PCV vaccine coverage
- Percent living in urban setting
- Population density
- Pre-term birth prevalence
- Sanitation quality
- Surrounding conflict
- Stunting prevalence
- Time to care seeking
- Total fertility rate
- Travel time to major city or settlement
- Travel time to nearest health care facility
- Under-five mortality rate
- Underweight prevalence
- Vaccine coverage equity
- Vaccination efficacy
- Vaccination schedule
- Vitamin A deficiency prevalence
- Vitamin A supplementation
- Vitamin A treatment
- Wasting prevalence

Section S4. Ranked list of indicators related to measles case fatality, with average rank.

1. Age (1.86)
2. MCV1 coverage (8.43)
3. Underweight prevalence (9.71)
4. Wasting prevalence (9.71)
5. Vitamin A treatment (13.29)
6. Travel time to nearest health facility (13.86)
7. MCV2 coverage (14.00)
8. Level of health care available (14.57)
9. ORT/S for measles-related diarrhea (14.86)
10. Antibiotic use for measles-related pneumonia (15.14)
11. Time to care seeking (15.71)
12. Vitamin A deficiency prevalence (15.71)
13. Stunting prevalence (18.00)
14. Measles incidence (19.57)
15. Surrounding conflict (20.00)
16. Health expenditure per capita (20.14)
17. Access to ICU (20.29)
18. Measles attack rate (20.43)
19. Under-5 mortality (20.71)
20. LRI prevalence (21.00)
21. Diarrheal disease prevalence (21.29)
22. Average household size (21.71)
23. Outbreak setting indicator (23.00)
24. Vitamin A supplementation (23.29)
25. HIV prevalence (23.71)
26. Travel time to nearest city or settlement (23.71)
27. Population density (25.43)
28. Sanitation quality (25.43)
29. HIV treatment prevalence / ART prevalence (25.86)
30. Maternal (measles) vaccination coverage (26.00)
31. Preterm birth prevalence (26.00)
32. TFR / average children per woman (26.86)
33. Percent living in urban setting (27.00)
34. Proxy for maternal antibody dynamics (27.71)
35. Proxy for vaccine coverage equity (28.71)
36. PCV vaccine coverage (30.29)
37. Educational attainment (30.71)
38. Vaccination efficacy (32.14)
39. Ambient air pollution (32.71)
40. Malaria prevalence (33.71)
41. De-worming frequency (34.43)
42. Vaccination schedule (36.71)

Section S5. PRISMA checklists

Section S5a. PRISMA compliance checklist

Section and Topic	Item #	Checklist item	Location where item is reported
TITLE			
Title	1	Identify the report as a systematic review.	Title; identified as literature review
ABSTRACT			
Abstract	2	See the PRISMA 2020 for Abstracts checklist.	Supplementary Information Section 5b
INTRODUCTION			
Rationale	3	Describe the rationale for the review in the context of existing knowledge.	Introduction
Objectives	4	Provide an explicit statement of the objective(s) or question(s) the review addresses.	Introduction
METHODS			
Eligibility criteria	5	Specify the inclusion and exclusion criteria for the review and how studies were grouped for the syntheses.	Methods (Literature Review subsection)
Information sources	6	Specify all databases, registers, websites, organisations, reference lists and other sources searched or consulted to identify studies. Specify the date when each source was last searched or consulted.	Methods (Literature Review subsection)
Search strategy	7	Present the full search strategies for all databases, registers and websites, including any filters and limits used.	Methods (Literature Review subsection); Supplementary Information Section 6
Selection process	8	Specify the methods used to decide whether a study met the inclusion criteria of the review, including how many reviewers screened each record and each report retrieved, whether they worked independently, and if applicable, details of automation tools used in the process.	Methods (Literature Review subsection); Contributions
Data collection process	9	Specify the methods used to collect data from reports, including how many reviewers collected data from each report, whether they worked independently, any processes for obtaining or confirming data from study investigators, and if applicable, details of automation tools used in the process.	Methods (Literature Review subsection); Contributions
Data items	10a	List and define all outcomes for which data were sought. Specify whether all results that were compatible with each outcome domain in each study were sought (e.g.	Methods (Literature Review subsection)

Section and Topic	Item #	Checklist item	Location where item is reported
		for all measures, time points, analyses), and if not, the methods used to decide which results to collect.	
	10b	List and define all other variables for which data were sought (e.g. participant and intervention characteristics, funding sources). Describe any assumptions made about any missing or unclear information.	Methods (Literature Review subsection)
Study risk of bias assessment	11	Specify the methods used to assess risk of bias in the included studies, including details of the tool(s) used, how many reviewers assessed each study and whether they worked independently, and if applicable, details of automation tools used in the process.	Contributions
Effect measures	12	Specify for each outcome the effect measure(s) (e.g. risk ratio, mean difference) used in the synthesis or presentation of results.	Results
Synthesis methods	13a	Describe the processes used to decide which studies were eligible for each synthesis (e.g. tabulating the study intervention characteristics and comparing against the planned groups for each synthesis (item #5)).	N/A as no synthesis was performed
	13b	Describe any methods required to prepare the data for presentation or synthesis, such as handling of missing summary statistics, or data conversions.	N/A as no synthesis was performed
	13c	Describe any methods used to tabulate or visually display results of individual studies and syntheses.	N/A as no synthesis was performed
	13d	Describe any methods used to synthesize results and provide a rationale for the choice(s). If meta-analysis was performed, describe the model(s), method(s) to identify the presence and extent of statistical heterogeneity, and software package(s) used.	N/A as no synthesis was performed
	13e	Describe any methods used to explore possible causes of heterogeneity among study results (e.g. subgroup analysis, meta-regression).	N/A as no synthesis was performed
	13f	Describe any sensitivity analyses conducted to assess robustness of the synthesized results.	N/A as no sensitivity analyses were conducted
Reporting bias assessment	14	Describe any methods used to assess risk of bias due to missing results in a synthesis (arising from reporting biases).	Methods (Literature Review subsection)
Certainty assessment	15	Describe any methods used to assess certainty (or confidence) in the body of evidence for an outcome.	N/A as no synthesis was performed
RESULTS			
Study	16a	Describe the results of the search and selection process,	Results; Figure 2

Section and Topic	Item #	Checklist item	Location where item is reported
selection		from the number of records identified in the search to the number of studies included in the review, ideally using a flow diagram.	
	16b	Cite studies that might appear to meet the inclusion criteria, but which were excluded, and explain why they were excluded.	N/A; no studies met this criteria
Study characteristics	17	Cite each included study and present its characteristics.	Results; Supplementary Information Sections 7-8
Risk of bias in studies	18	Present assessments of risk of bias for each included study.	Results; Supplementary Information Sections 7-8
Results of individual studies	19	For all outcomes, present, for each study: (a) summary statistics for each group (where appropriate) and (b) an effect estimate and its precision (e.g. confidence/credible interval), ideally using structured tables or plots.	Results
Results of syntheses	20a	For each synthesis, briefly summarise the characteristics and risk of bias among contributing studies.	N/A as no synthesis was performed
	20b	Present results of all statistical syntheses conducted. If meta-analysis was done, present for each the summary estimate and its precision (e.g. confidence/credible interval) and measures of statistical heterogeneity. If comparing groups, describe the direction of the effect.	N/A as no statistical analysis was conducted
	20c	Present results of all investigations of possible causes of heterogeneity among study results.	N/A as no synthesis was performed
	20d	Present results of all sensitivity analyses conducted to assess the robustness of the synthesized results.	N/A as no sensitivity analyses were conducted
Reporting biases	21	Present assessments of risk of bias due to missing results (arising from reporting biases) for each synthesis assessed.	N/A as no synthesis was performed
Certainty of evidence	22	Present assessments of certainty (or confidence) in the body of evidence for each outcome assessed.	N/A as no synthesis was performed
DISCUSSION			
Discussion	23a	Provide a general interpretation of the results in the context of other evidence.	Discussion
	23b	Discuss any limitations of the evidence included in the review.	Discussion

Section and Topic	Item #	Checklist item	Location where item is reported
	23c	Discuss any limitations of the review processes used.	Discussion
	23d	Discuss implications of the results for practice, policy, and future research.	Discussion
OTHER INFORMATION			
Registration and protocol	24a	Provide registration information for the review, including register name and registration number, or state that the review was not registered.	This review was not registered
	24b	Indicate where the review protocol can be accessed, or state that a protocol was not prepared.	A protocol was not prepared
	24c	Describe and explain any amendments to information provided at registration or in the protocol.	N/A
Support	25	Describe sources of financial or non-financial support for the review, and the role of the funders or sponsors in the review.	Declarations of Interest; Contributions
Competing interests	26	Declare any competing interests of review authors.	Declarations of Interest
Availability of data, code and other materials	27	Report which of the following are publicly available and where they can be found: template data collection forms; data extracted from included studies; data used for all analyses; analytic code; any other materials used in the review.	Results; Supplementary Information Sections 7-8

Section S5b. PRISMA compliance abstract checklist

Section and Topic	Item #	Checklist item	Reported (Yes/No)
TITLE			
Title	1	Identify the report as a systematic review.	Yes
BACKGROUND			
Objectives	2	Provide an explicit statement of the main objective(s) or question(s) the review addresses.	Yes
METHODS			
Eligibility criteria	3	Specify the inclusion and exclusion criteria for the review.	Yes
Information sources	4	Specify the information sources (e.g. databases, registers) used to identify studies and the date when each was last searched.	Yes
Risk of bias	5	Specify the methods used to assess risk of bias in the included studies.	Yes
Synthesis of results	6	Specify the methods used to present and synthesise results.	N/A
RESULTS			
Included studies	7	Give the total number of included studies and participants and summarise relevant characteristics of studies.	Yes
Synthesis of results	8	Present results for main outcomes, preferably indicating the number of included studies and participants for each. If meta-analysis was done, report the summary estimate and confidence/credible interval. If comparing groups, indicate the direction of the effect (i.e. which group is favoured).	Yes
DISCUSSION			
Limitations of evidence	9	Provide a brief summary of the limitations of the evidence included in the review (e.g. study risk of bias, inconsistency and imprecision).	Yes
Interpretation	10	Provide a general interpretation of the results and important implications.	Yes
OTHER			
Funding	11	Specify the primary source of funding for the review.	Declarations of Interest
Registration	12	Provide the register name and registration number.	This review was not registered.

Section S6. Full list of indicator-specific search terms for systematic review

("educational attainment" OR "education" OR "educat*" OR "school"
OR "urban" OR "crowding" OR "dens*"
OR "conflict" OR "unrest" OR "war" OR "disobedience" OR “state of emergency” OR “pariah state”
OR "travel to health facility" OR “distance to health facility”
OR "care seeking" OR “care-seeking”
OR "proximity to city" OR "travel time to city" OR “distance to city”
OR "stunting" OR "malnourished" OR “malnutrition”
OR "underweight"
OR "vitamin A supplementation"
OR "vitamin A deficien*"
OR "wasting"
OR "ICU" OR "intensive care"
OR "health expenditure" OR "health spending" OR "spending" OR “healthcare per capita”
OR "health care quality" OR "healthcare quality" OR “health care access” OR “healthcare access”
OR "under 5 mortality" OR "under-5 mortality" OR "under five mortality" OR "under-five mortality" OR "infant mortality" OR "child mortality" OR "under 5 death" OR "under-5 death" OR "under five death" OR "under-five death"
OR "air pollution" OR "smog"
OR "antibiotic" OR "pneumonia"
OR "household"
OR "de-worming" OR "deworming"
OR "diarrhea" OR “rotavirus”
OR "HIV" OR "human immunodeficiency virus" OR "acquired immunodeficiency syndrome" OR "AIDS"
OR "antiretroviral therapy" OR "ART"
OR "malaria" OR "plasmodium falciparum" OR "plasmodium vivax"
OR "lower respiratory infection" OR "LRI"
OR "oral rehydration”
OR "pneumococcal vaccine" OR "pneumococcal conjugate vaccine"
OR "pre-term birth" OR "preterm birth" OR “low birthweight” OR “low birth weight”
OR "total fertility rate" OR "average children per women" OR “average number of children per woman”
OR “births per woman” OR “parity”
OR "measles attack rate" OR "measles transmission"
OR "measles incidence"
OR "maternal antibody"
OR "maternal measles vaccination" OR "maternal measles immunity" OR "maternal measles vaccine"
OR (("second dose" OR "MCV2" OR "first dose" OR "MCV1" OR "vaccination" OR "vaccine" OR "immunization") AND “coverage”)
OR "equity"
OR "vaccination efficacy" OR "immunization efficacy"
OR "vaccination schedule" OR "immunization schedule" OR "recommended age of vaccination" OR "recommended age of immunization" OR “dosing schedule”
OR "vitamin A treatment"
)
AND “measles”
AND (“case fatality” OR “CFR” OR “fatality” OR “mortality” OR “morbid*” OR “comorbid*” OR “sever*” OR “complicat*” OR “risk” OR “secondary outcome” OR “death”)

Section S7. Studies containing evidence of an association between measles CFR and specified indicator

Lead Author	Title	Country	Publication Year	Indicator(s)	Measurement of association	Has sample size > 100?	Clinical trial?	Indicates lab confirmation of measles cases?	Provides definition of acute death attributable to measles?	Measure of association adjusted for confounding?	Quality score
Aaby, P.	Overcrowding and intensive exposure as determinants of measles mortality	Guinea-Bissau	1984	Average household size	RR: 1.9 [1.2 – 3.0]	X	--	--	X	--	3
Aaby, P.	Measles mortality, state of nutrition, and family structure: a community study from Guinea-Bissau	Guinea-Bissau	1983	Average household size	Chi-squared p<0.01	--	--	--	X	--	2
Aaby, P.	The survival benefit of measles immunization may not be explained entirely by the prevention of measles disease: a community study from rural Bangladesh	Bangladesh	2003	MCV1 coverage	vaccine efficacy against measles death: 95% [79%- 99%]	--	--	--	X	--	2
Ahmed, P. A.	Review of childhood measles admissions at the National Hospital, Abuja	Nigeria	2010	Underweight prevalence	chi-squared p=0.01	--	--	--	--	--	1
Alwar, A. J.	The effect of protein energy malnutrition on morbidity and mortality due to measles at Kenyatta National Hospital, Nairobi (Kenya)	Kenya	1992	Malnutrition	RR: 3.77 [1.85–7.66]	X	--	--	--	--	2
Aurangzeb, B.	Clinical outcome in children hospitalized with complicated measles	Pakistan	2005	MCV1 coverage	OR: 8.40 [1.00–71.84]	X	--	--	--	--	2
Aurangzeb, B.	Risk factors for mortality among admitted children with complications of measles in Pakistan: An observational study	Pakistan	2021	Stunting prevalence	OR: 6.8 [3.24–14.26]	X	--	--	X	--	3
				Underweight prevalence	OR: 2.93 [1.44–5.93]						
				MCV2 coverage	RR (against unvaccinated): 7.0 [2.03–24.01] and RR (against only receiving one dose): 5.73 [1.49–22.07])						
Avila-Figueroa, C.	Complications in children with measles	Mexico	1990	Malnutrition	RR: 2.47 [1.1–5.52]	X	--	X	X	--	4
Barclay, A. J.	Vitamin A supplements and mortality related to measles: a randomised clinical trial	Tanzania	1987	Underweight prevalence	RR: 3.94 [1.69–9.21]	X	--	--	X	--	3
Bhuiya, A.	Measles case fatality among the under-fives: a multivariate analysis of risk factors in a rural area of Bangladesh	Bangladesh	1987	Educational attainment	OR: 2.11 [1.06–4.19]	X	--	--	X	--	3
Burström, B.	Child mortality in Stockholm during 1885-1910: the impact of household size and number of children in the family on the risk of death from measles	Sweden	1999	Average household size	RR (siblings): 2.9 [1.6–5.4] and RR (households with more than four persons): 1.9 [1.3–2.8]	X	--	--	--	--	2
Choudhry, V. P.	Effect of protein energy malnutrition on the immediate outcome of measles	Afghanistan	1987	Malnutrition	RR: 14.66 [5.46–39.36]	X	--	--	--	--	2
Clemens, J. D.	Measles vaccination and childhood mortality in rural Bangladesh	Bangladesh	1988	Educational attainment	OR (head of household): 1.32 [1.07–1.63] and OR (mothers): 1.72 [1.36–2.19]	X	--	--	--	--	2
Coetzee, S.	Measles in a South African paediatric intensive care unit: again!	South Africa	2014	Underweight prevalence	RR: 2.77 [1.38–5.55]	--	--	X	--	--	2
				HIV prevalence	RR: 2.29 [1.24–4.20]						
Commey, J. O.	Measles in Ghana--1973-1982	Ghana	1984	Malnutrition	RR: 2.02 [1.63–2.51]	X	--	--	--	--	2
Courtright, P.	Abnormal vitamin A cytology and mortality in infants aged 9 months and less with measles	Malawi	2002	Vitamin A deficiency prevalence	RR: 4.00 [1.21–13.33]	X	--	--	--	--	2

Dollimore, N.	Measles incidence, case fatality, and delayed mortality in children with or without vitamin A supplementation in rural Ghana	Ghana	1997	Underweight prevalence	OR: 2.5 [1.3–5.1]	X	--	--	--	X	3
				MCV1 coverage	RR: 1.72 [1.04–2.84]						
Dzeyie, K. A.	Measles outbreak investigation at Indo-Myanmar border, Longding District, Arunachal Pradesh, India, 2017	India	2021	Vitamin A treatment	chi-squared p=0.0351	--	--	X	X	--	3
Fetuga, M. B.	A ten-year study of measles admissions in a Nigerian teaching hospital	Nigeria	2007	Malnutrition	RR: 7.33 [1.62–33.16]	X	--	--	--	--	2
				MCV1 coverage	Association noted (p=0.033)						
Gignoux, E.	Risk factors for measles mortality and the importance of decentralized case management during an unusually large measles epidemic in eastern Democratic Republic of Congo in 2013	Democratic Republic of the Congo	2018	Travel time to nearest health care facility	RR: 2.2 [1.0–4.7]	X	--	X	X	--	4
				MCV1 coverage	RR: 0.3 [0.1–0.9]						
				MCV2 coverage	RR: 0.2 [0.1–0.3]						
Gutu, M. A.	Epidemiology of measles in Oromia region, Ethiopia, 2007-2016	Ethiopia	2020	MCV1 coverage	OR: 1.55 [1.14–2.11]	X	--	X	--	--	3
Hussey, G. D.	A randomized, controlled trial of vitamin A in children with severe measles	South Africa	1990	Vitamin A treatment	OR: 0.21 [0.05–0.94]	X	X	--	--	--	3
Hussey, G. D.	Routine high-dose vitamin A therapy for children hospitalized with measles	South Africa	1993	Vitamin A treatment	OR: 0.36 [0.18–0.70]	X	X	--	--	--	3
Jeena, P. M.	Infectious diseases at the paediatric isolation units of Clairwood and King Edward VIII Hospitals, Durban. Trends in admission and mortality rates (1985-1996) and the early impact of HIV (1994-1996)	South Africa	1998	HIV prevalence	RR: 129.62 [40.12–412.64]	X	--	X	--	--	3
Joshi, A. B.	Measles deaths in Nepal: estimating the national case-fatality ratio	Nepal	2009	Surrounding conflict	OR: 15.8 [3.4–73.4]	X	--	X	X	--	4
				Stunting prevalence	RR: 5.34 [2.31–12.36]						
				MCV1 coverage	RR: 3.7 [2.0–6.7]						
				Vitamin A treatment	RR: 3.09 [1.69–5.67]						
Lagunju, I. A.	Measles in Ibadan: a continuous scourge	Nigeria	2005	Underweight prevalence	RR: 2.23 [1.17–4.26]	X	--	--	--	--	2
le Roux, D. M.	South African measles outbreak 2009- 2010 as experienced by a paediatric hospital	South Africa	2012	Malnutrition	Association noted	X	--	X	--	X	4
				HIV prevalence	OR: 7.55 [2.27–25.12]						
Lee, C. T.	Increase in Infant Measles Deaths During a Nationwide Measles Outbreak-Mongolia, 2015-2016	Mongolia	2019	Travel time to nearest city or settlement	RR: 1.9 [1.3–2.8]	X	--	X	X	--	4
Madhulika	Vitamin A supplementation in post-measles complications	India	1994	Malnutrition	Chi-squared p=0.0156	X	--	--	--	--	2
Malina, R. M.	Epidemiologic transition in an isolated indigenous community in the Valley of Oaxaca, Mexico	Mexico	2008	Under-5 mortality rate	Correlation noted	--	--	--	--	--	1
Meteke, S.	Delivering infectious disease interventions to women and children in conflict settings: a systematic review	Various	2020	Surrounding conflict	Qualitative	--	--	--	--	--	1
Mgone, J. M.	Control measures and the outcome of the measles epidemic of 1999 in the Eastern Highlands Province	Papua New Guinea	2000	MCV1 coverage	chi-squared p=0.0423	X	--	--	--	--	2
Moss, W. J.	Measles still has a devastating impact in unvaccinated populations	Various	2007	Surrounding conflict	Qualitative	--	--	--	--	--	1
				Malnutrition	Qualitative						
				Vitamin A deficiency	Qualitative						

				MCV1 coverage	Qualitative						
Moss, W. J.	HIV type 1 infection is a risk factor for mortality in hospitalized Zambian children with measles	Zambia	2008	Educational attainment	RR: 2.15 [1.11–4.17]	X	--	X	--	--	3
				HIV prevalence	RR: 2.95 [1.83–4.74]						
Moss, W. J.	Measles	Various	2017	Malnutrition	Qualitative	--	--	--	--	--	1
				MCV1 coverage	Qualitative						
Murhekar, M. V.	Measles case fatality rate in Bihar, India, 2011-12	India	2014	Educational attainment	RR: 6.23 [1.48–26.21]	X	--	X	X	--	4
				Vitamin A treatment	RR: 0.14 [0.03–0.61]						
Nandy, R.	Case-fatality rate during a measles outbreak in eastern Niger in 2003	Niger	2006	Average household size	RR: 1.82 [1.22 – 2.71]	X	--	X	X	--	4
Nayir, T.	Effects of immunization program on morbidity and mortality rates of vaccine-preventable diseases in Turkey	Turkey	2020	MCV coverage	Association noted	--	--	--	--	--	1
Ndikuyeze, A.	Priorities in global measles control: report of an outbreak in N'Djamena, Chad	Chad	1995	Educational attainment	Qualitative	--	--	--	X	--	2
Nojilana, B.	Estimating the burden of disease attributable to vitamin A deficiency in South Africa in 2000	South Africa	2007	Vitamin A deficiency prevalence	RR: 1.86 [1.32–2.59]	--	--	--	--	--	1
Oshitani, H.	Measles infection in hospitalized children in Lusaka, Zambia	Zambia	1995	MCV coverage/vaccination status	RR: 0.4 [0.19–0.83]	X	--	--	--	--	2
Oshitani, H.	Measles case fatality by sex, vaccination status, and HIV-1 antibody in Zambian children	Zambia	1996	HIV prevalence	RR: 3.35 [1.95–5.76]	X	--	--	X	--	3
Rey, M.	Impact of measles in France	France	1983	Level of health care available	Qualitative	--	--	--	--	--	1
Rosero-Bixby, L.	Socioeconomic development, health interventions and mortality decline in Costa Rica	Costa Rica	1991	Under-5 mortality rate	Correlation noted	--	--	--	--	--	1
Salama, P.	Malnutrition, measles, mortality, and the humanitarian response during a famine in Ethiopia	Ethiopia	2001	Surrounding conflict	Qualitative	X	--	--	--	--	2
				Under-5 mortality rate	Correlation noted						
Samb, B.	Decline in measles case fatality ratio after the introduction of measles immunization in rural Senegal	Senegal	1997	MCV1 coverage	Association noted (p=0.038)	--	--	--	X	--	2
Samsi, T. K.	Risk factors for severe measles	Indonesia	1992	Malnutrition	RR: 2.48 [1.4–4.39]	X	--	--	--	--	2
Sepúlveda, J.	Improvement of child survival in Mexico: the diagonal approach	Mexico	2006	Under-5 mortality rate	Correlation noted	--	--	--	--	--	1
Spencer, H. C.	Impact on mortality and fertility of a community-based malaria control programme in Saradidi, Kenya	Kenya	1987	Under-5 mortality rate	Correlation noted	--	--	--	--	--	1

Section S8. Studies containing non-significance evidence of an association between measles CFR and specified indicator

Lead Author	Title	Country	Publication Year	Indicator(s)	Has sample size > 100?	Clinical trial?	Indicates lab confirmation of measles cases?	Provides definition of acute death attributable to measles?	Measure of association adjusted for confounding?	Quality score
Aaby, P.	Vaccinated children get milder measles infection: a community study from Guinea-Bissau	Guinea-Bissau	1986	MCV1 coverage	X	--	--	--	--	2
Aaby, P.	Measles mortality, state of nutrition, and family structure: a community study from Guinea-Bissau	Guinea-Bissau	1983	Malnutrition, educational attainment	--	--	--	X	--	2

Aaby, P.	Measles incidence, vaccine efficacy, and mortality in two urban African areas with high vaccination coverage	Guinea-Bissau	1990	MCV1 coverage	--	--	--	--	--	1
Aaby, P.	Overcrowding and intensive exposure as determinants of measles mortality	Guinea-Bissau	1984	Average household size, malnutrition	X	--	--	X	--	3
Adu, F. D.	Measles outbreak in Ibadan: clinical, serological and virological identification of affected children in selected hospitals	Nigeria	1997	MCV1 coverage	--	--	X	--	--	2
Ahmed, P. A.	Review of childhood measles admissions at the National Hospital, Abuja	Nigeria	2010	MCV1 coverage	--	--	--	--	--	1
Ananthakrishnan, S.	Vitamin A and post measles complications	India	1993	Vitamin A deficiency prevalence	X	--	--	--	--	2
Ariyasriwatana, C.	Severity of measles: a study at the Queen Sirikit National Institute of Child Health	Thailand	2004	HIV prevalence	X	--	--	--	--	2
Arya, L. S.	Spectrum of complications of measles in Afghanistan: a study of 784 cases	Afghanistan	1987	Malnutrition	X	--	--	--	--	2
Aurangzeb, B.	Clinical outcome in children hospitalized with complicated measles	Pakistan	2005	Malnutrition, proportion living in urban setting	X	--	--	--	--	2
Barclay, A. J.	Vitamin A supplements and mortality related to measles: a randomised clinical trial	Tanzania	1987	Vitamin A treatment	X	--	--	X	--	3
Coakley, K. J.	A review of measles admissions and deaths in the paediatric ward of Goroka Base Hospital during 1989	Papua New Guinea	1991	Preterm birth, vitamin A treatment	--	--	--	--	--	1
Coetzee, S.	Measles in a South African paediatric intensive care unit: again!	South Africa	2014	Antibiotic use, vitamin A treatment, PCV coverage	--	--	X	--	--	2
Courtright, P.	Abnormal vitamin A cytology and mortality in infants aged 9 months and less with measles	Malawi	2002	MCV1 coverage	X	--	--	--	--	2
Coutsoudis, A.	Vitamin A supplementation reduces measles morbidity in young African children: a randomized, placebo-controlled, double-blind trial	South Africa	1991	Vitamin A treatment	--	X	--	--	--	2
Dollimore, N.	Measles incidence, case fatality, and delayed mortality in children with or without vitamin A supplementation in rural Ghana	Ghana	1997	Average household size, educational attainment	X	--	--	--	X	3
Donadel, M.	Risk factors for measles deaths among children during a Nationwide measles outbreak-Romania, 2016-2018	Romania	2021	Antibiotic use, PCV coverage, preterm birth, vitamin A treatment, MCV1 coverage, malnutrition	X	--	X	X	X	5
Fischer, P. R.	Measles in Zaire: 1987	Democratic Republic of the Congo	1988	Malnutrition, MCV1 coverage	X	--	--	--	--	2
Gignoux, E.	Risk factors for measles mortality and the importance of decentralized case management during an unusually large measles epidemic in eastern Democratic Republic of Congo in 2013	Democratic Republic of the Congo	2018	HH size	X	--	X	X	--	4
Gutu, M. A.	Epidemiology of measles in Oromia region, Ethiopia, 2007-2016	Ethiopia	2020	Proportion living in urban setting	X	--	X	--	--	3
Hull, H. F.	Increased measles mortality in households with multiple cases in the Gambia, 1981	Gambia	1988	Average household size	X	--	--	--	--	2
Joshi, A. B.	Measles deaths in Nepal: estimating the national case-fatality ratio	Nepal	2009	Average household size	X	--	X	X	--	4
Julien, M.	Changing patterns in pediatric mortality, Maputo Central Hospital, Mozambique, 1980-1990	Mozambique	1995	Malaria prevalence	X	--	--	--	--	2
Khoo, A.	Measles--an experience in Sandakan Hospital, Sabah, 1990	Malaysia	1994	Malnutrition	X	--	--	--	--	2
Koster, F. T.	Mortality among primary and secondary cases of measles in Bangladesh	Bangladesh	1988	Malnutrition	X	--	X	--	--	3

Lagunju, I. A.	Measles in Ibadan: a continuous scourge	Nigeria	2005	MCV1 coverage	X	--	--	--	--	2
le Roux, D. M.	South African measles outbreak 2009- 2010 as experienced by a paediatric hospital	South Africa	2012	MCV1 coverage	X	--	X	--	X	4
Lee, C. T.	Increase in Infant Measles Deaths During a Nationwide Measles Outbreak-Mongolia, 2015-2016	Mongolia	2019	Antibiotic use, malnutrition, MCV1 coverage, vitamin A treatment	X	--	X	X	--	4
Mafigiri, R.	Risk factors for measles death: Kyegegwa District, western Uganda, February-September, 2015	Uganda	2017	Malnutrition	--	--	X	--	--	2
Markowitz, L. E.	Vitamin A levels and mortality among hospitalized measles patients, Kinshasa, Zaire	Democratic Republic of the Congo	1989	Wasting prevalence	X	--	--	--	--	1
Moss, W. J.	Prospective study of measles in hospitalized, human immunodeficiency virus (HIV)-infected and HIV-uninfected children in Zambia	Zambia	2002	HIV prevalence	X	--	X	--	--	3
Munir, M.	Measles and its problems. A clinical analysis of hospitalized patients under 5 years of age	Indonesia	1982	Malnutrition	X	--	--	--	--	2
Nandy, R.	Case-fatality rate during a measles outbreak in eastern Niger in 2003	Niger	2006	Vitamin A treatment, travel time to nearest health care facility	X	--	X	X	X	5
Ogaro, F. O.	Effect of vitamin A on diarrhoeal and respiratory complications of measles	Kenya	1993	Vitamin A treatment	X	X	--	--	--	3
Sension, M. G.	Measles in hospitalized African children with human immunodeficiency virus	Democratic Republic of the Congo	1988	HIV prevalence	X	--	X	--	--	3
Smedman, L.	Nutritional status and measles: a community study in Guinea-Bissau	Guinea-Bissau	1983	Malnutrition	X	--	--	X	--	3

Section S9. Indicators with significant evidence of an association with highest quality score from contributing studies

Mechanism	Indicator	Highest quality score
Health system access and care-seeking behaviors	Educational attainment	4
	Percent living in urban settings	2
	Surrounding conflict	4
	Travel time to major city or settlement	4
	Travel time to nearest health care facility	4
Health system quality	Level of health care available	1
	Under-five mortality rate	2
Measles control and epidemiology	First-dose coverage of measles-containing vaccine (MCV1)	4
	Second-dose coverage of measles-containing vaccine (MCV2)	4
	Vitamin A treatment	4
Nutritional status	Stunting prevalence	4
	Underweight prevalence	3
	Vitamin A deficiency prevalence	2
	Wasting prevalence	4
Risk of secondary infection	Average household size	4
	Human immunodeficiency virus (HIV) prevalence	4
